# Comparison of intravitreal injection techniques with and without a speculum using eyelid retraction with a cotton-tipped applicator

**DOI:** 10.1186/s40942-025-00739-5

**Published:** 2025-10-29

**Authors:** Hanny Rui Qi Chen, Gabriel Costa de Andrade, Lucas Kenji Arimori, Fernando Kruglensky Lerner

**Affiliations:** 1 Department of Ophthalmology, Irmandade da Santa Casa de Misericórdia de São Paulo, São Paulo, Brazil; 2 Faculdade de Ciências Médicas da Santa Casa de São Paulo, São Paulo , Brazil

**Keywords:** Intravitreal injection, Eyelid speculum, Pain, Cotton swab, Visual analog scale, Eyelid retraction

## Abstract

**Background:**

Intravitreal injection (IVI) is a widely performed ophthalmic procedure with generally low complication rates, but repeated applications are often required due to the short half-life of anti-VEGF agents. Pain management during IVI is critical for patient quality of life and adherence to treatment (Andrade and Carvalho in Arq Bras Oftalmol 2015;78:27–31). Prior studies have suggested that eyelid specula may increase discomfort, while alternative retraction methods could improve patient tolerance.

**Main body:**

In this prospective, randomized clinical trial, 106 patients were assigned to receive IVI either with an eyelid speculum or with cotton-swab–assisted eyelid retraction. Immediately after the procedure, patients rated pain using a 10-cm visual analog scale (VAS). Complications were also recorded. The mean VAS pain score was 2.34 ± 1.83 (95% CI: 1.83–2.85) in the speculum group and 1.37 ± 1.58 (95% CI: 0.93–1.80) in the cotton-swab group. The mean difference of 0.97 points was statistically significant (ANOVA, *p* = 0.0041). The most frequent complication was subconjunctival hemorrhage (9.4%), with no cases of endophthalmitis, retinal detachment, or lens touch.

**Conclusion:**

Under the conditions of this study, IVI performed without a speculum using cotton-swab eyelid retraction was associated with lower reported pain and comparable complication rates. This technique may enhance patient comfort and treatment adherence, particularly for those requiring multiple injections each year, although further validation is warranted.

## Background

Intravitreal injection (IVI) of anti-vascular endothelial growth factor (anti-VEGF) agents is one of the most frequently performed procedures worldwide for conditions such as age-related macular degeneration [2], diabetic retinopathy [3], and retinal vein occlusion [4]. Although safe, the need for repeated injections highlights the importance of patient comfort. This study evaluates whether cotton-swab eyelid retraction can reduce discomfort compared with standard speculum use.

## Methods

This randomized, prospective trial enrolled 106 patients at Santa Casa de Misericórdia de São Paulo between January and August 2023. Sample size was calculated using G*Power 3.1 with assumptions of a 1.0-point difference in VAS, α = 0.05, power = 80%. Patients were randomized 1:1 by computer-generated sequence, allocation concealed in opaque envelopes. Exclusion criteria included severe ocular surface disease, recent analgesic/sedative use, and uncooperative behavior. Interventions followed a standardized protocol using either a speculum or cotton-swab retraction [5–8]. Pain was assessed immediately post-procedure by an independent evaluator using a 10-cm VAS [9]. Complications were recorded. Analyses included one-way ANOVA with effect sizes and chi-square tests for categorical variables.

## Results

The mean pain score was 2.34 ± 1.83 (95% CI: 1.83–2.85) in the speculum group and 1.37 ± 1.58 (95% CI: 0.93–1.80) in the cotton-swab group. The difference of 0.97 was statistically significant (*p* = 0.0041) [10]. Subconjunctival hemorrhage occurred in 9.4% of cases, with no severe complications. Pain did not differ significantly between naïve and previously injected patients (*p* = 0.83) [11].

**Fig. 1:**
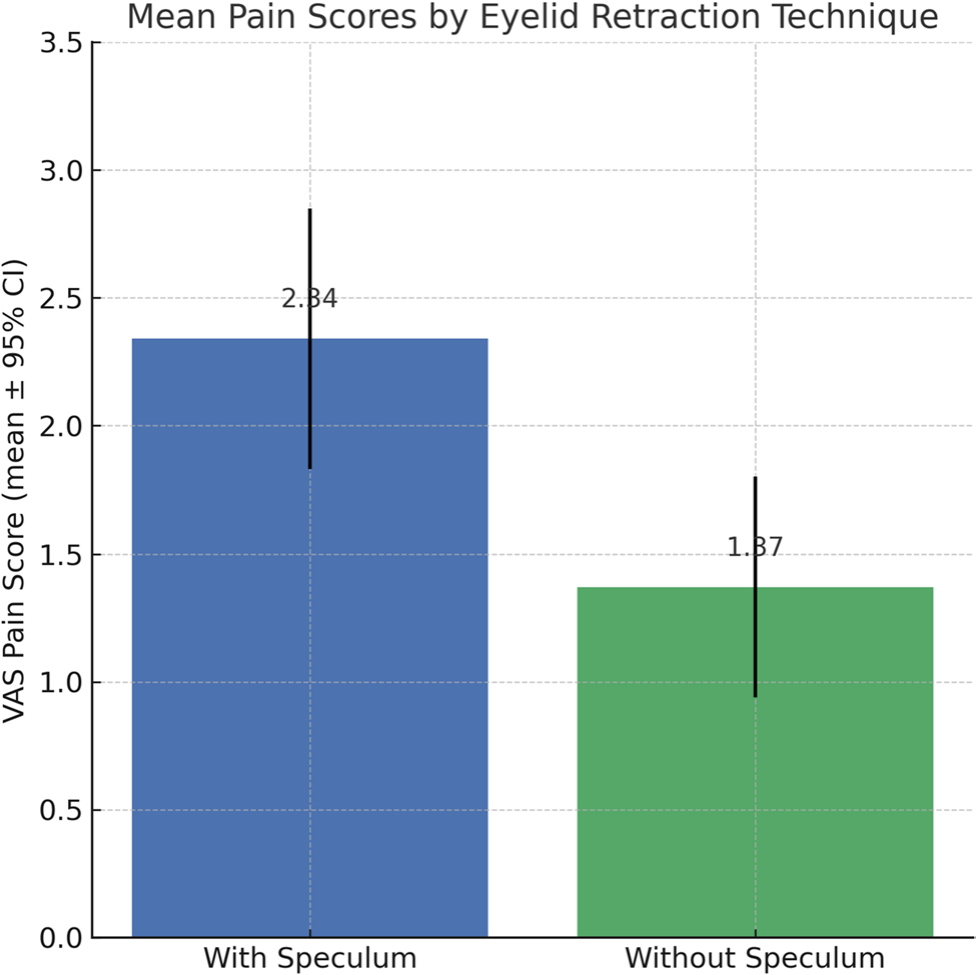
Mean VAS pain score according to eyelid retraction technique

Mean pain scores between groups are presented in Fig. 1.

Baseline patient demographics and clinical characteristics are summarized in Table 1.

**Table 1:** Patient demographics, diagnoses, injection site, prior injection history, and complications. Values are n (%)

Characteristic	With Speculum (*n* = 52)	Without Speculum (*n* = 54)	Total (*N* = 106)
Sex			
Male	28 (26.4%)	25 (23.6%)	53 (50.0%)
Female	24 (22.6%)	29 (27.4%)	53 (50.0%)
Primary diagnosis			
Age-related macular degeneration	7 (6.6%)	5 (4.7%)	12 (11.3%)
Central retinal vein occlusion	10 (9.4%)	3 (2.8%)	13 (12.3%)
Diabetic retinopathy	35 (33.0%)	46 (43.4%)	81 (76.4%)
Injection site			
Right eye	19 (17.9%)	26 (24.5%)	45 (42.5%)
Left eye	33 (31.1%)	28 (26.4%)	61 (57.5%)
Previous injections			
None	4 (3.8%)	4 (3.8%)	8 (7.6%)
1–5 injections	31 (29.2%)	28 (26.4%)	59 (55.7%)
6–10 injections	10 (9.4%)	8 (7.5%)	18 (17.0%)
> 10 injections	7 (6.6%)	3 (2.8%)	10 (9.4%)
Complications			
None	46 (43.4%)	50 (47.2%)	96 (90.6%)
Subconjunctival hemorrhage	6 (5.7%)	4 (3.8%)	10 (9.4%)

## Discussion

This trial demonstrates that cotton-swab eyelid retraction is associated with lower pain compared to speculum-assisted IVI [12, 13], without increasing complication rates [14]. Limitations include exclusion of uncooperative patients and the predominance of diabetic patients, which may bias pain perception. Although vitreous reflux was less frequent in the cotton-swab group, the study was not powered to assess this outcome. Efficiency claims were removed, as procedure time was not measured. Environmental impact depends on local context: disposable cotton swabs reduce sterilization needs, while reusable specula may also be sustainable [15, 16]. Our findings are consistent with prior literature and add strength by using a randomized prospective design [17–19]. Minor procedure-related events, such as subconjunctival hemorrhage, occurred at comparable rates to those reported in previous patient-based studies [20–22].

## Conclusions

The cotton-swab eyelid retraction method was significantly less painful and equally safe compared with the speculum technique. This approach may improve adherence in patients requiring multiple injections, though larger multicenter trials are needed.

## Data Availability

The datasets are available from the corresponding author upon reasonable request.

## Abbreviations

IVI: Intravitreal injection
VAS: Visual analog scale
VEGF: Vascular endothelial growth factor
CI: Confidence interval
DR: Diabetic retinopathy
